# Supplementary material for: Mechano-osmotic signals control chromatin state and fate transitions in pluripotent stem cells
Source: Nat Cell Biol. 2025 Sep 29;27(10):1757–70. doi: 10.1038/s41556-025-01767-x (PMC12527910; doi:10.1038/s41556-025-01767-x)
Supplement: Supplementary file 1 — Reporting Summary [file 41556_2025_1767_MOESM1_ESM.pdf]

nature portfolio

Corresponding author(s): Sara A. Wickström, Yekaterina A. Miroshnikova

Last updated by author(s): July 17th 2025

Reporting Summary

Nature Portfolio wishes to improve the reproducibility of the work that we publish. This form provides structure for consistency and transparency in reporting. For further information on Nature Portfolio policies, see our Editorial Policies and the Editorial Policy Checklist.

Statistics

For all statistical analyses, confirm that the following items are present in the figure legend, table legend, main text, or Methods section.

n/a	Confirmed	
☐	☒	The exact sample size (*n*) for each experimental group/condition, given as a discrete number and unit of measurement
☐	☒	A statement on whether measurements were taken from distinct samples or whether the same sample was measured repeatedly
☐	☒	The statistical test(s) used AND whether they are one- or two-sided<br>*Only common tests should be described solely by name; describe more complex techniques in the Methods section.*
☒	☐	A description of all covariates tested
☐	☒	A description of any assumptions or corrections, such as tests of normality and adjustment for multiple comparisons
☐	☒	A full description of the statistical parameters including central tendency (e.g. means) or other basic estimates (e.g. regression coefficient) AND variation (e.g. standard deviation) or associated estimates of uncertainty (e.g. confidence intervals)
☐	☒	For null hypothesis testing, the test statistic (e.g. *F*, *t*, *r*) with confidence intervals, effect sizes, degrees of freedom and *P* value noted<br>*Give P values as exact values whenever suitable.*
☒	☐	For Bayesian analysis, information on the choice of priors and Markov chain Monte Carlo settings
☒	☐	For hierarchical and complex designs, identification of the appropriate level for tests and full reporting of outcomes
☒	☐	Estimates of effect sizes (e.g. Cohen's *d*, Pearson's *r*), indicating how they were calculated

Our web collection on statistics for biologists contains articles on many of the points above.

Software and code

Policy information about availability of computer code

Data collection	Andor Fusion software (spinning disc confocal microscopy, version 2.3.0.44)<br>Leica Application Suite X (confocal microscopy, version 2.0.0.14332)<br>JPK SPM Control Software (version 5)<br>Nikon Software (NIS-Elements AR 5.41.01)<br>Zeiss ZEN Software (Zeiss ZEN version 3.7),
Data analysis	Zeiss ZEN Software (Zeiss ZEN version 3.7)<br>Cellpose (version 2.2.2)<br>Fiji (version 2.0.0)<br>JPK Data Processing Software (Bruker Nano, version 5)<br>Python (3.8)<br>R (v4.2.2)<br>Cell Ranger Arc (v.1.1.2)<br>scanpy (v1.8.2)<br>SCENIC+ (pyscenic v0.11.2)<br>FastP (v0.23.2)<br>bwa-mem2 (v.2.2.1)<br>sambamba (v 1.0.1)<br>deepTools (v 3.5.4)<br>DESeq2 (v1.34.0)<br>Perseus (v1.6.15)

scipy (v1.11.4)
MaxQuant (v2.4.0)
Zeiss ZEN Software (Zeiss ZEN version 3.7)
GraphPad Prism software (GraphPad, version 9)

For manuscripts utilizing custom algorithms or software that are central to the research but not yet described in published literature, software must be made available to editors and reviewers. We strongly encourage code deposition in a community repository (e.g. GitHub). See the Nature Portfolio guidelines for submitting code & software for further information.

Data

Policy information about availability of data

All manuscripts must include a data availability statement. This statement should provide the following information, where applicable:

- Accession codes, unique identifiers, or web links for publicly available datasets
- A description of any restrictions on data availability
- For clinical datasets or third party data, please ensure that the statement adheres to our policy

Sequencing datasets are available at GEO
Project accession: GSE26809:

Proteomic datasets are available at PRIDE
Project accession: PXD052588

Data will be freely available upon publication. All other data supporting the findings of this study are available from the corresponding author on reasonable request.

Human research participants

Policy information about studies involving human research participants and Sex and Gender in Research.

Reporting on sex and gender	N/A
Population characteristics	N/A
Recruitment	N/A
Ethics oversight	N/A

Note that full information on the approval of the study protocol must also be provided in the manuscript.

Field-specific reporting

Please select the one below that is the best fit for your research. If you are not sure, read the appropriate sections before making your selection.

☒ Life sciences ☐ Behavioural & social sciences ☐ Ecological, evolutionary & environmental sciences

For a reference copy of the document with all sections, see nature.com/documents/nr-reporting-summary-flat.pdf

Life sciences study design

All studies must disclose on these points even when the disclosure is negative.

Sample size	Sample size was determined based on previous experience, published literature, or to specific requirements of a given technique. Sample size for each experiment is indicated in figure legends
Data exclusions	Sequencing results were filtered by quality using fastp and files with low quality were excluded from subsequent analyses.
Replication	All experiments were performed using at least three biological replicates. Number of replicates for each experiment is indicated in the corresponding figure legend. Several steps were taken to ensure the reproducibility of experimental findings and key results were confirmed using complementary experimental approaches.
Randomization	Samples were not randomized, randomization was not relevant as samples were grouped according to treatment.

Blinding	Blinding was not used. This was not meaningfuly as automated software algorithms were used for unbiased quantification of staining intensities and sequencing data.

Reporting for specific materials, systems and methods

We require information from authors about some types of materials, experimental systems and methods used in many studies. Here, indicate whether each material, system or method listed is relevant to your study. If you are not sure if a list item applies to your research, read the appropriate section before selecting a response.

Materials & experimental systems

n/a	Involved in the study
☐	☒ Antibodies
☐	☒ Eukaryotic cell lines
☒	☐ Palaeontology and archaeology
☒	☐ Animals and other organisms
☒	☐ Clinical data
☒	☐ Dual use research of concern

Methods

n/a	Involved in the study
☒	☐ ChIP-seq
☒	☐ Flow cytometry
☒	☐ MRI-based neuroimaging

Antibodies

Antibodies used	OCT3/4 (Santa-Cruz Biotechnology, sc-5279; 1:1000), Brachyury (R&D Systems, AF2085; 1:1000), GATA6 (AF1700, RnD Systems; 1:200), NANOG (D73G4, Cell Signaling Technologies; 1:200), LAMINB1 (66095-1-Ig, Proteintech; 1:200), LaminB1 (Cell Signaling 9087; 1:1000), Pax6 (Invitrogen, #42-6600; 1:1000), SOX1 (R&D Systems, AF3369; 1:200), SOX7 (R&D Systems, AF1924; 1:1000), YAP1 (Santa Cruz sc-101199; 1:200), CBX2 (Thermo Fisher PA-582812; 1:800), p38 MAPK phospho Thr180/Tyr182 (Cell signaling 4511, 1:1000), p38 MAPK (Cell Signaling 9212, 1:1000), p44/42 MAPK phospho-Erk1/2 (Cell Signaling 4376, 1:2000), p44/42 MAPK Erk1/2 (Cell Signaling 4695, 1:1000), RNApol2 PS2 (Abcam ab5095; 1:5000), H3K27ac (Abcam, ab4729). Alexa Fluor 488, 568, 594 and 647 conjugated antibodies (all from Invitrogen) were used as secondary antibodies at 1:500 dilution.
Validation	All antibodies are well characterized and widely used in the literature. They were applied according to datasheet instructions or previously published protocols with respect to staining and Western Blotting.

Eukaryotic cell lines

Policy information about cell lines and Sex and Gender in Research

Cell line source(s)	Allen Institute LMNB1 mTagRFP (AICS-0034 cl.62); Allen Institute SOX2 mEGFP (AICS-0074 cl.26); dCas9-KRAB TagBFP (AICS-0090 cl.391); dCas9-TagBFP-KRAB Halotag-YAP1 knockin, dCas9-TagBFP-KRAB CBX2-GFP knockin
Authentication	Cell lines from the Allen Institute were rigorously authenticated by the supplier. Halotag-YAP1 and CBX2-GFP insertions were authenticated by specific primers.
Mycoplasma contamination	Cell cultures were routinely confirmed mycoplasma-negative.
Commonly misidentified lines (See ICLAC register)	*Name any commonly misidentified cell lines used in the study and provide a rationale for their use.*
