# Supplementary figures and images for: Mechano-osmotic signals control chromatin state and fate transitions in pluripotent stem cells
Source: Nat Cell Biol. 2025 Sep 29;27(10):1757–70. doi: 10.1038/s41556-025-01767-x (PMC12527910; doi:10.1038/s41556-025-01767-x)

Extended Data 5d

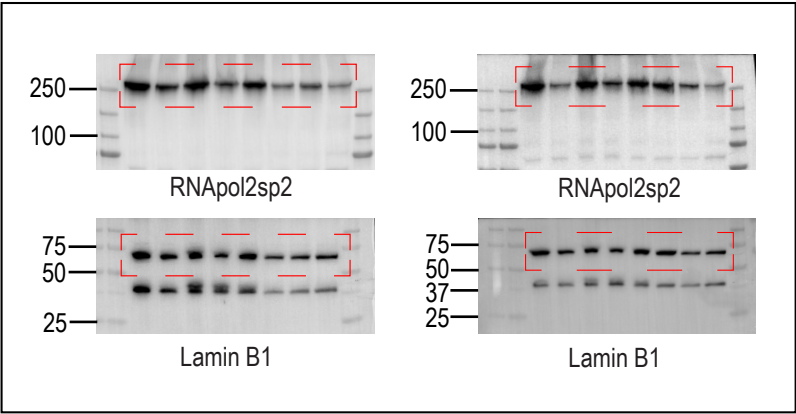

Extended Data 5e

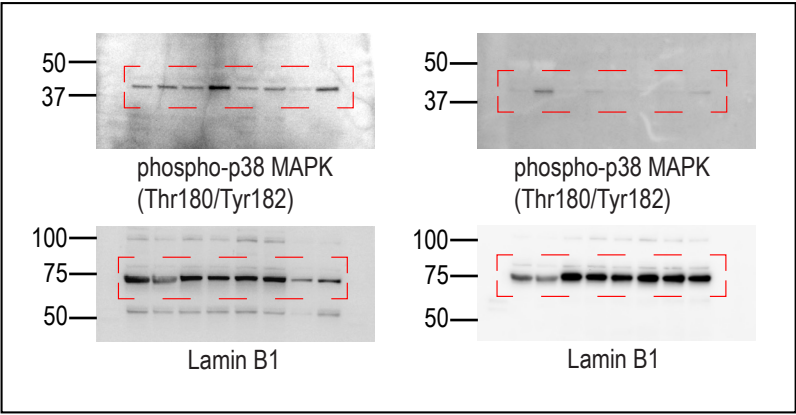

Supplement: Supplementary file 13 — Full-length, unprocessed gels for Extended Data Fig. 5d,e. [file 41556_2025_1767_MOESM13_ESM.pdf]
